# Quantitative volumetric analysis of the Golgi apparatus following X-ray irradiation by super-resolution 3D-SIM microscopy

**DOI:** 10.1007/s00795-020-00277-z

**Published:** 2021-01-26

**Authors:** Takahiro Oike, Yuki Uchihara, Tiara Bunga Mayang Permata, Soehartati Gondhowiardjo, Tatsuya Ohno, Atsushi Shibata

**Affiliations:** 1 Department of Radiation Oncology, Gunma University Graduate School of Medicine, 3-39-22, Showa-machi, Maebashi, Gunma 371-8511 Japan; 2 Gunma University Heavy Ion Medical Center, 3-39-22, Showa-machi, Maebashi, Gunma 371-8511 Japan; 3 Signal Transduction Program, Gunma University Initiative for Advanced Research (GIAR), 3-39-22, Showa-machi, Maebashi, Gunma 371-8511 Japan; 4 Department of Radiation Oncology, Faculty of Medicine Universitas Indonesia-Dr. Cipto Mangunkusumo Hospital, Jl. P. Diponegoro No. 71, Jakarta, 10430 Indonesia

**Keywords:** Golgi, Ionizing radiation, Super-resolution microscopy, 3D-SIM, RCAS1

## Abstract

To obtain quantitative volumetric data for the Golgi apparatus after ionizing radiation (IR) using super-resolution three-dimensional structured illumination (3D-SIM) microscopy. Normal human retinal pigment epithelial (RPE) cells were irradiated with X-rays (10 Gy), followed by immunofluorescence staining of the Golgi marker RCAS1. 3D-SIM imaging was performed using DeltaVision OMX version 4 and SoftWoRx 6.1. Polygon rendering and spot signal identification were performed using Imaris 8.1.2. Differences between groups were assessed by Welch’s *t* test. RCAS1 signals in untreated cells were located adjacent to nuclei and showed a reticular morphology. Upon IR, the area of RCAS1 signals expanded while retaining the reticular morphology. Polygon rendering imaging revealed that the volume of RCAS1 at 48 h post-IR was greater than that for unirradiated cells (93.7 ± 19.0 μm^3^ vs. 33.0 ± 4.2 μm^3^, respectively; *P* < 0.001): a 2.8-fold increase. Spot signal imaging showed that the number of RCAS1 spot signals post-IR was greater than that for unirradiated cells [3.4 ± 0.8 (× 10^3^) versus 1.3 ± 0.2 (× 10^3^), respectively; *P* < 0.001]: a 2.7-fold increase. This is the first study to report quantitative volumetric data of the Golgi apparatus in response to IR using super-resolution 3D-SIM microscopy.

**Supplementary Information:**

The online version contains supplementary material available at 10.1007/s00795-020-00277-z.

## Introduction

Ionizing radiation (IR) triggers various biological consequences in mammalian cells. While nuclear responses to IR (e.g., DNA repair and signaling, cell cycle checkpoint arrest, and regulation of gene expression) have been studied extensively [1, 2], the responses of cytoplasmic organelles to IR remain unclear. The Golgi apparatus is a major cytoplasmic organelle involved in the sorting, packaging, and modifying of proteins synthesized in the endoplasmic reticulum [3]. In addition, recent studies demonstrate a role for the Golgi apparatus in various cellular processes, e.g., the DNA damage response (DDR) [4], migration [5], metabolism [6], and autophagy [7].

Historically, the morphology of the Golgi apparatus has been investigated using electron microscopy [3], which shows that the Golgi apparatus in untreated cells is located at the periphery of the nucleus and comprises stacks of narrow or slightly dilated ternae [3]. Upon IR, the Golgi apparatus appears to break up and scatter throughout the cytoplasm [3]. Meanwhile, studies using confocal fluorescent microscopy describe the post-IR morphology of the Golgi apparatus as “fragmented”, “dispersed”, or “disorganized” [3, 4, 8]. However, these morphological characteristics of the post-IR architecture of the Golgi apparatus are not backed up by quantitative and volumetric data, regardless of the modality used.

Recent technological advancements have improved the resolution of fluorescence microscopy, achieving ~ 100 nm resolution [9]. This class of advanced fluorescence microscopy comprises three-dimensional structured illumination (3D-SIM), stimulated emission depletion (STED), and photo-activated localization microscopy (PALM). Of these, 3D-SIM achieves a resolution of approximately 100 nm along the *x*- and *y*-axes, and approximately 300 nm along the *z*-axis. Compared with STED and PALM, 3D-SIM has a great advantage with respect to 3D-analysis. In addition, 3D-SIM can be used to analyze samples prepared using conventional immunofluorescence staining methods [10]. To date, we have examined foci of γH2AX and of replication protein A, and we have measured the 3D distribution of the sites of DNA double-strand breaks (DSBs) undergoing homologous recombination-associated resection within huge and complex DSB lesions in cells irradiated with high linear-energy-transfer carbon ions [11].

The objective of the present study was to use super-resolution 3D-SIM imaging to obtain quantitative volumetric data for the Golgi apparatus labeled with the Golgi marker RCAS1 in response to IR [12, 13].

## Materials and methods

### Cell culture and irradiation

Retinal pigment epithelial (RPE) cells were obtained from Clontech Laboratories, Inc. (Palo Alto, CA, USA; product name, hTERT RPE-1; product number, cat no. C4000-1). Representative images of cell morphology before and after IR, taken under a phase-contrast microscope, are shown in Online Resource 1. RPE cells were cultured in the Dulbecco’s Modified Eagle’s Medium/Nutrient Mixture F-12 (Fujifilm; Tokyo, Japan) supplied with 10% fetal calf serum (Sigma-Aldrich; St. Louis, MO, USA) and 1 × Penicillin–Streptomycin-l-Glutamine Solution (Fujifilm). X-ray irradiation was performed using a MX-160Labo (mediXtec; Chiba, Japan: 160 kVp, 1.07 Gy/min, and 3.0 mA).

### Immunofluorescence staining

RPE cells were seeded on cover glasses 1 s (Matsunami; Osaka, Japan) 24 h prior to the experiment to obtain cells in the exponential growth phase. Cells in G2 phase were identified by CENPF staining. To identify cells in S phase, EdU was added 30 min prior to X-ray irradiation. Next, cells were fixed for 10 min in 3% paraformaldehyde–2% sucrose and permeabilized for 3 min with 0.2% TritonX-100-phosphate buffered saline (PBS). Cells were washed twice with PBS and incubated at 37 °C for 30 min with the primary antibody in Solution A (TOYOBO; Osaka, Japan). Cells were then washed with PBS and incubated at 37 °C for 30 min with secondary antibodies conjugated to Alexa Fluor 488/594 in 2% bovine serum albumin (Sigma-Aldrich)/PBS containing 0.1 mg/mL 4′,6-diamidino-2-phenylindole, dihydrochloride (DAPI; Roche, Mannheim, Germany). Subsequently, cells were stained with Click-iT™ EdU. After an additional wash with PBS, cover glasses were mounted in Vectashield (Vector Laboratories; Burlingame, CA, USA). The following primary antibodies were used: anti-RCAS1 (1:400, #12290; Cell Signaling Technology, Danvers, MA, USA) and anti-CENPF (1:400, #610768; BD Biosciences, Franklin Lakes, NJ, USA). The following kit was used for EdU staining: Click-iT™ EdU Cell Proliferation Kit for Imaging, Alexa Fluor™ 647 dye (Thermo Fisher Scientific; Waltham, MA, USA).

### Conventional imaging by immunofluorescence microscopy

**Fig. 1:**
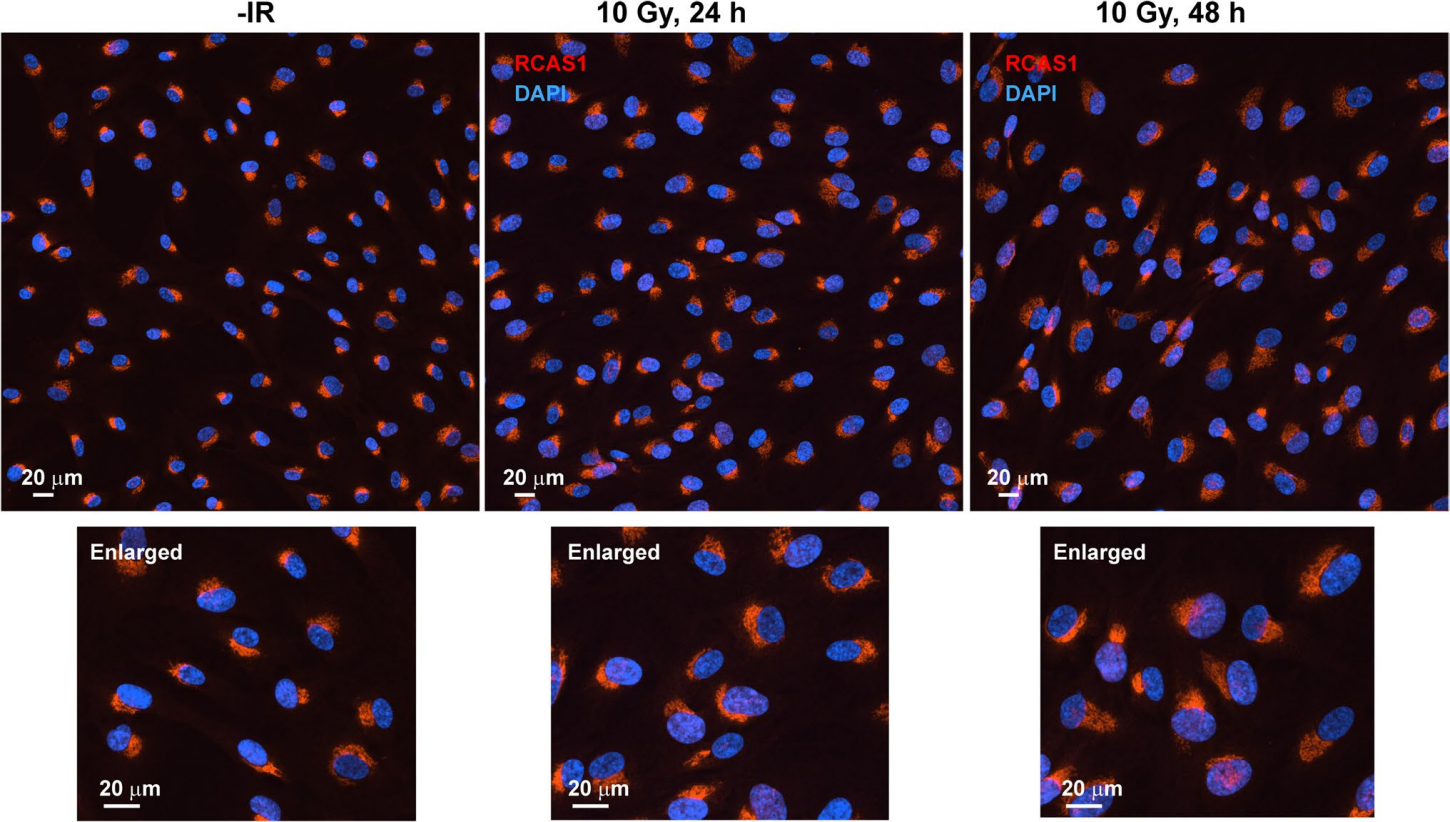
Ionizing radiation-induced changes cell morphology and in the RCAS1 signal. RPE cells were irradiated with 10 Gy X-rays and fixed at the indicated times. Cells were stained with an anti-RCAS1 antibody and DAPI. Enlarged images of the boxes are shown at the bottom of each image

**Fig. 2:**
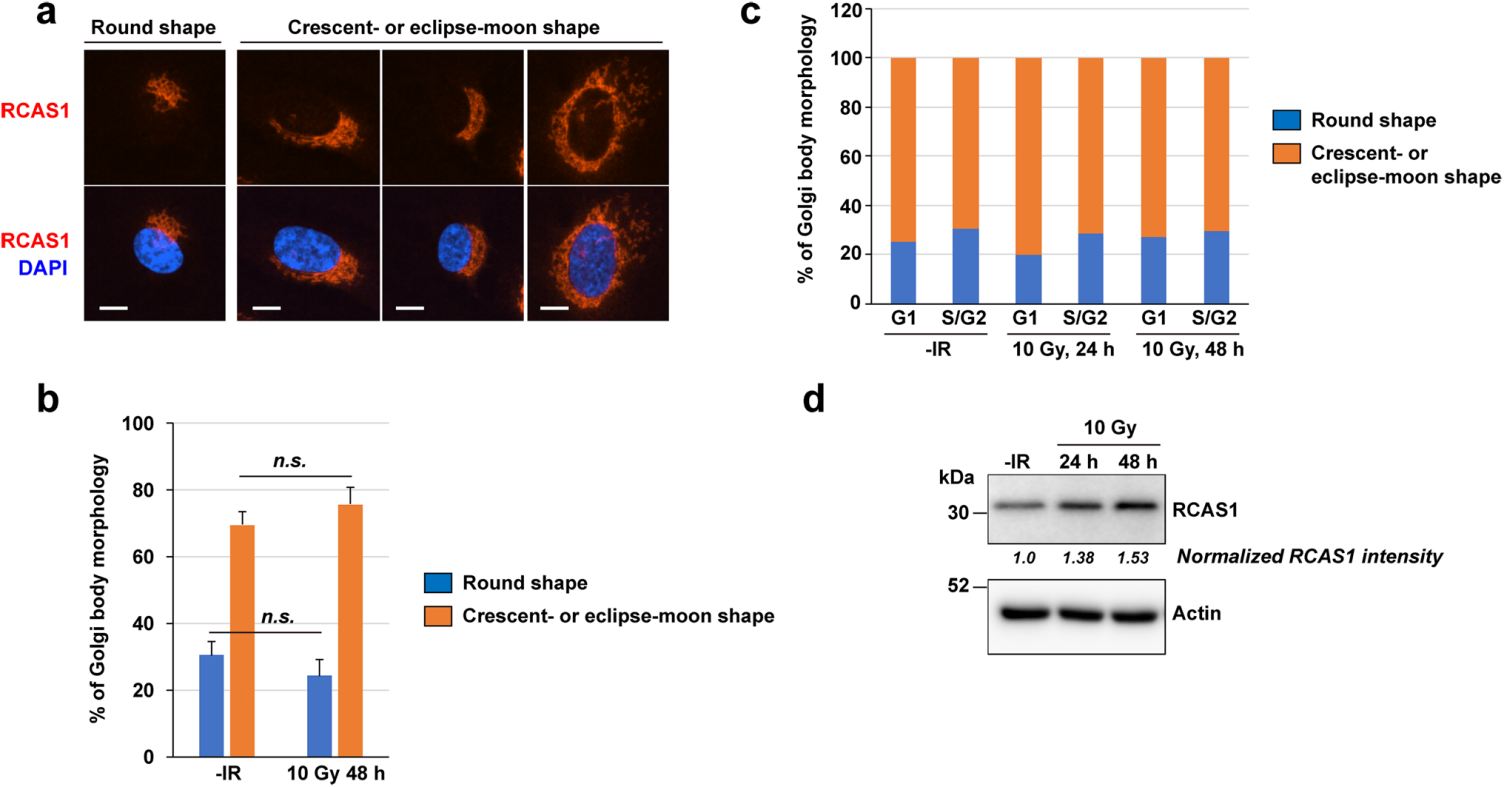
Morphology of RCAS1 at different phases of the cell cycle. **a** Representative images of RCAS1, categorized according to the shape of the RCAS1 signal (“round” and “crescent-or eclipse-moon shaped”) after exposure of cells to 10 Gy X-rays. Scale bar 10 µm. **b** Percentage of cells showing each type of RCAS1 signal morphology after exposure to X-rays.** c** Percentage of cells in G1 or S/G2 phase showing each type of RCAS1 signal morphology after exposure to X-rays. **d** Expression of RCAS1 in the presence/absence of X-rays was examined by immunoblotting. Ratio of RCAS1/actin expression after X-rays exposure, normalized to the signal in unirradiated cells

The representative images shown in Figs. 1 and 2 were taken using a Nikon ECLIPSE Ni microscope equipped with a 40× objective lens, a DS-Qi2 camera, and NIS-Elements D imaging software (Nikon, Tokyo, Japan).

### 3D-SIM microscopy and volume rendering

3D-SIM was performed using a microscope system (DeltaVision OMX version 4; GE Healthcare, UK) equipped with 405 and 568 nm solid-state lasers. The detailed settings for the DeltaVision OMX were described previously [11]. Optical z-sections were separated by 0.125 μm. Laser lines at 405 and 568 nm were used for 3D-SIM acquisition. Typical exposure times were between 60 and 80 ms, and the power of each laser was adjusted to achieve optimal intensities of between 10,000 and 30,000 counts in a raw image at 15-bit dynamic range; the lowest possible power was used to minimize photobleaching. Multichannel imaging was achieved by sequential acquisition of wavelengths using separate cameras. Raw 3D-SIM images were processed and reconstructed using the DeltaVision OMX SoftWoRx 6.1 software package (GE Healthcare). The channels were aligned carefully using alignment parameters obtained from control measurements from image registration calibration slides and 0.1 μm TetraSpeck™ Fluorescent Microspheres (Molecular Probes; Eugene, OR, USA). The lateral and axial resolutions of the 3D-SIM images were > 135 ± 5 nm and > 350 ± 15 nm, respectively. Resolution details were described previously [14–16].

To measure the volume of RCAS1, 3D polygon rendering was created by the *Surface* module of Imaris 8.1.2. (Oxford Instruments; Abingdon, UK). The volume of the *Surface* images was recorded as the volume of RCAS1. The *Spot* module was used to identify individual spot signals generated by RCAS1. Following selection of polygon rendering regions using the *Masked* module, the number of spots and the intensity of each spot within a given surface were measured.

### Statistical analysis

Differences in numerical variables between two groups were assessed using Welch’s *t* test and R software [17]. *P* values < 0.05 were considered statistically significant. Box plots and scatter plots were created using the ggplot2 package in R.

## Results

First, we used conventional fluorescence microscopy with low magnification settings to obtain an overall picture of morphological changes in the Golgi apparatus in response to IR (Fig. 1). We used RPE (a normal human cell line) cells because they have an intact gene status, including the DDR [18–20]. To visualize the localization of the Golgi apparatus, we selected RCAS1 as a Golgi marker [12, 13]. In untreated cells, RCAS1 signals were located adjacent to nuclei (Fig. 1, left panel). At 24 or 48 h post-IR with 10 Gy, the area of the RCAS1 signals expanded, although the juxtanuclear position was retained (Fig. 1, middle and right panels). RCAS1 signals showed crescent- or eclipse-moon morphologies in approximately 70% of cells examined; a round morphology was observed in the remaining 30% (Fig. 2a, b). These percentages remained consistent, regardless of IR (Fig. 2b) or cell cycle phase (Fig. 2c; Online Resource 2). Interestingly, western blot analysis showed a mild increase in expression of cellular RCAS1 post-IR (Fig. 2d), indicating that IR not only changes the spatial distribution of the Golgi apparatus but also induces volumetric changes, with an increase in RCAS1 protein expression.

**Fig. 3:**
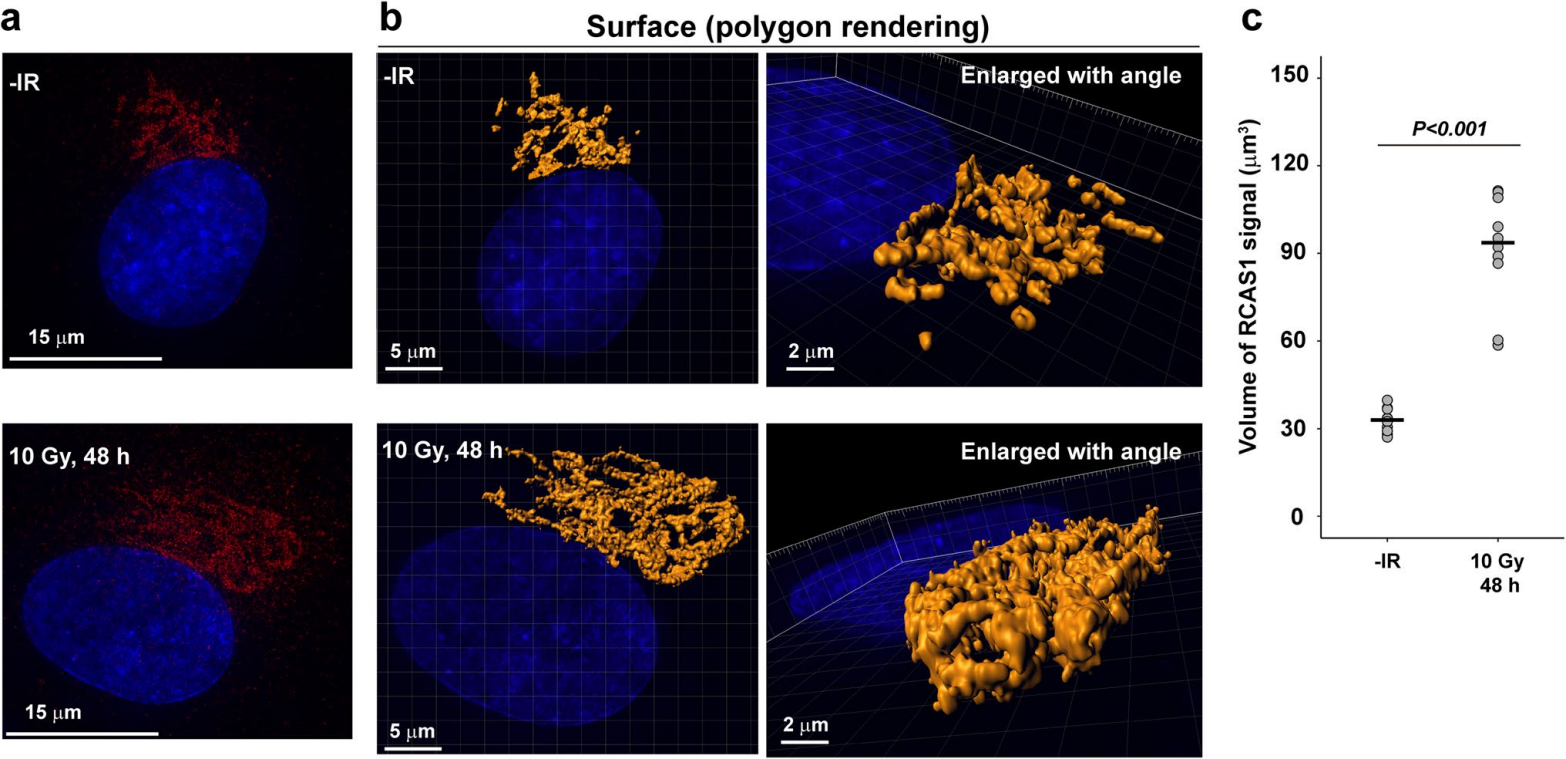
Visualization of RCAS1 signals in X-ray-irradiated cells by super-resolution imaging using 3D-SIM. **a** Representative images of RCAS1 and DAPI are shown. RPE cells were irradiated with 10 Gy X-rays, fixed 48 h later, and stained with an anti-RCAS1 antibody and DAPI. Images are taken using the 3D-SIM setting in DeltaVision OMX version 4. **b** Surface polygon images of RCAS1 were generated by Imaris 8.1.2. **c** Volume of RCAS1 was measured after generation of polygon rendering using imaging software Imaris 8.1.2. RPE cells were irradiated with 10 Gy X-rays, fixed 48 h later, and stained with an anti-RCAS1 antibody and DAPI. Statistical significance was determined using Welch’s *t* test. ***P < 0.001

**Fig. 4:**
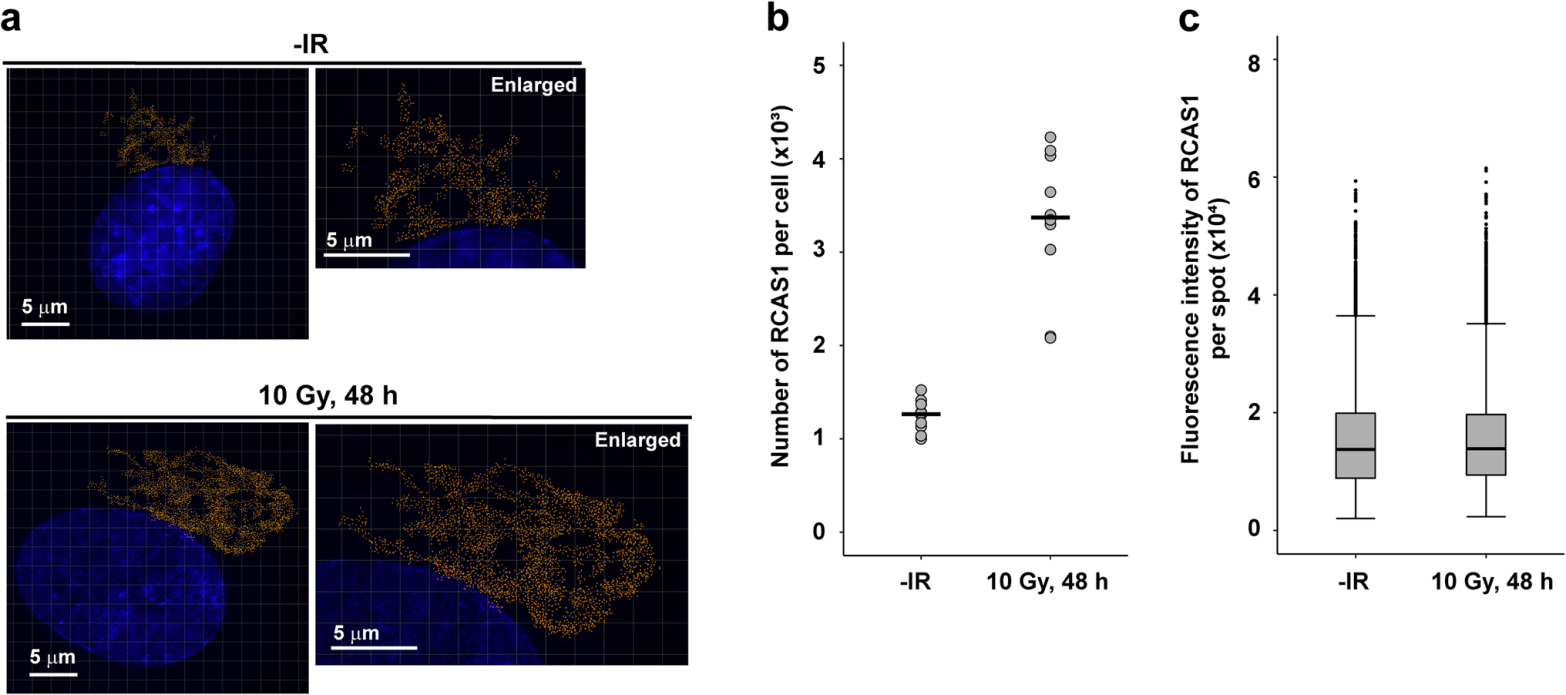
Quantification of the number of RCAS1 spots and signal intensity in irradiated cells. **a** Cells were fixed 48 h after X-ray exposure and then stained with an anti-RCAS1 antibody and DAPI. To measure the number of individual RCAS1 spot signals, the center of the fluorescence intensity signal was identified and visualized by Imaris 8.1.2. Number of RCAS1 spots (**b**) and maximal signal intensity of each spot (**c**) was measured by Imaris. Statistical significance was determined using Welch’s *t* test. ***P < 0.001

To further investigate volumetric changes in the Golgi apparatus in response to IR, we analyzed RCAS1 signals using super-resolution modes of a 3D-SIM and the DeltaVision OMX system. The super-resolution RCAS1 signals (Fig. 3a) were translated into polygon rendering images (Fig. 3b and Online Resource 3, 4) and the volumes were measured (Fig. 3c). To uniformly quantify changes in Golgi volume after IR, we selected round-shaped Golgi apparatus (Fig. 2a) and analyzed their volume (Fig. 3c). The volume of RCAS1 at 48 h post-IR was significantly greater than that in untreated cells (93.7 ± 19.0 μm^3^ vs. 33.0 ± 4.2 μm^3^, respectively; *P* < 0.001): a 2.8-fold increase (Fig. 3c). To analyze the distribution of RCAS1 proteins within the Golgi apparatus in response to IR, we evaluated the number and intensity of super-resolution RCAS1 signals detected as individual spots (Fig. 4a). In line with the results from the polygon rendering images, the number of RCAS1 spot signals was significantly greater than that under untreated conditions [3.4 ± 0.8 (× 10^3^) vs. 1.3 ± 0.2 (× 10^3^), respectively; *P* < 0.001]: a 2.7-fold increase (Fig. 4b). By contrast, there was no significant difference in the fluorescence intensity of each RCAS1 spot signal between irradiated and unirradiated cells (Fig. 4c), suggesting that IR does not change the number of RCAS1 molecules in each spot. Taken together, the data indicate that IR not only changes the spatial distribution of the Golgi apparatus but also increases its volume.

## Discussion

To the best of our knowledge, this study is the first study to use super-resolution 3D-SIM microscopy to report Golgi morphology in DNA-damaged cells. Changes in Golgi morphology in response to IR have been investigated for decades. Studies based on immunofluorescence staining and conventional immunofluorescence microscopy describe the morphology of the Golgi apparatus in DNA-damaged cells (either treated with IR or chemotherapeutic reagents) as “fragmented”, “dispersed”, and “disorganized” [3, 4, 8]. This observation is consistent with those of studies using Golgi markers other than RCAS1 (e.g., GM130 [4], TGN38 [21], and p58 [3]). In the present study, we found that RCAS1 signals post-IR did not show simple fragmented or disorganized patterns when visualized by 3D-SIM imaging. Both polygon rendering (Fig. 3b) and spot signal imaging (Fig. 4a) revealed that RCAS1 signals in irradiated cells presented with a reticular morphology, which was broadly consistent with that observed in untreated cells. Importantly, 3D-SIM analysis showed that the fluorescence intensity in each RCAS1 spot did not change after IR. Although we could not count the number of RCAS1 molecules localized in each spot, the data show that the number of molecules per spot did not change markedly in response to DNA damage. In addition, 3D-SIM analysis revealed an increase (approximately threefold) in the volume of RCAS1 after IR, whereas immunoblot analysis revealed a ~ 1.5-fold increase in RCAS1 expression after IR. These data suggest that the size of Golgi cyst varies widely, without any change in the number or distribution of RCAS1. Hence, our study proposes that 3D-SIM is a useful tool for investigating the spatial distribution of the Golgi structure in response to cellular stresses such as DNA damage. Furthermore, the advantage of immunofluorescence is that it can visualize several proteins at the same time using different fluorescence probes (e.g., blue, green, red, and far-red). Although the immunofluorescence approach has some limitations, multi-color imaging will increase our understanding of the molecular interplay between Golgi functions controlled by multiple factors. However, our immunofluorescence approach targeting RCAS1 by 3D-SIM is unsuitable for visualizing the *cis*-, medial-, and *trans*-Golgi cisternae that are detectable by electron microscopy. Future research should combine experimental findings from electron microscopy and 3D-SIM, which will further increase our understanding of the functional role of the Golgi apparatus.

It is worth mentioning that RCAS1 is of interest to oncologists because of its predominant localization in the Golgi apparatus, i.e., it is thought that RCAS1 suppresses the activity of cytotoxic T-lymphocytes and natural killer cells by acting as a ligand for putative receptors expressed on these cells [22]. RCAS1 is expressed by lung, gastric, hepatic, breast, and uterine cancer cells [23–29]. Accordingly, RCAS1 expression in tumor specimens is a predictive biomarker for a worse prognosis [23, 24, 26–28]. From this perspective, the analytical methods used in this study can be used for high-resolution visualization of RCAS1 in the tumor microenvironment, in which cancer cells suppress antitumor immunity, thereby providing clues to identifying putative receptors for this molecule.

The present study has the following limitations. First, only RCAS1 was used to label the Golgi apparatus. Although multiple studies have used RCAS1 as “the Golgi marker” [12, 13], another study by Gelardi suggests that this protein is localized preferentially in the ER-Golgi intermediate compartment and in the *cis*-Golgi cisterna [30]. Therefore, analysis using additional Golgi markers, including TGN46 (*trans*-Golgi marker) and GM130 (*cis*-Golgi marker), is warranted to further elucidate the effect of X-rays on volumetric changes in the Golgi apparatus. Second, we did not identify the functional significance of the post-IR increase in the Golgi volume.

In summary, we report morphological changes in the Golgi apparatus in response to IR using super-resolution 3D-SIM microscopy. Upon IR, the Golgi apparatus increased in volume but retained its reticular morphology. Although further mechanistic studies are needed to fully elucidate the functional significance of this phenomenon, this study highlights a novel and powerful technique that can be used to investigate the structure and function of the Golgi apparatus.

## Supplementary Information

Below is the link to the electronic supplementary material.

Supplementary file1 (PDF 102 KB)

Supplementary file2 (PDF 84 KB)

Supplementary file3 (MPG 23352 KB)

Supplementary file4 (MPG 21866 KB)
