# Supplementary figures and images for: Quantitative volumetric analysis of the Golgi apparatus following X-ray irradiation by super-resolution 3D-SIM microscopy
Source: Med Mol Morphol. 2021 Jan 26;54(2):166–72. doi: 10.1007/s00795-020-00277-z (PMC8139881; doi:10.1007/s00795-020-00277-z)

## Online Resource 1. Representative image of RPE cells taken by phase-contrast microscopy

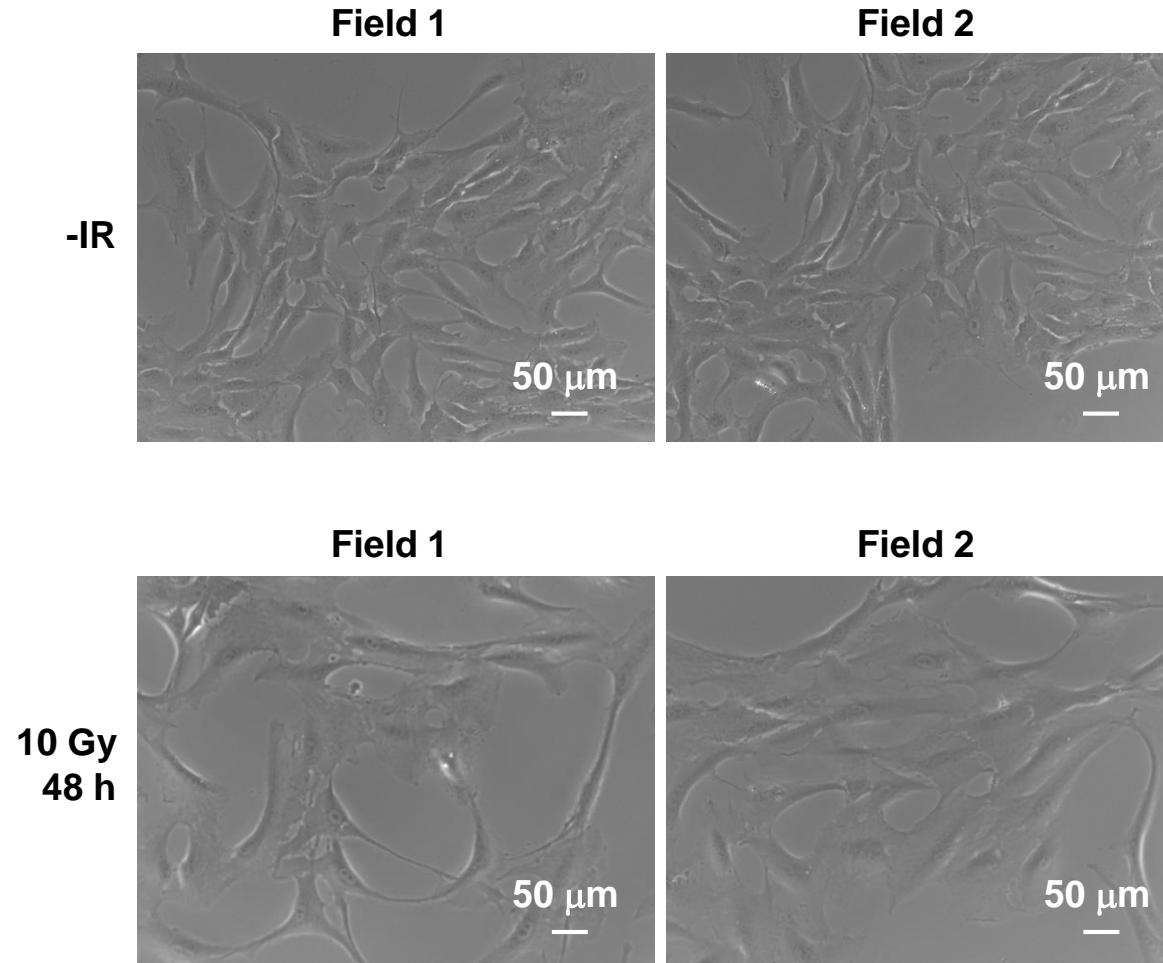

Supplement: Supplementary file 1 — Supplementary file1 (PDF 102 KB) [file 795_2020_277_MOESM1_ESM.pdf]
