# Supplementary material for: Quantitative volumetric analysis of the Golgi apparatus following X-ray irradiation by super-resolution 3D-SIM microscopy
Source: Med Mol Morphol. 2021 Jan 26;54(2):166–72. doi: 10.1007/s00795-020-00277-z (PMC8139881; doi:10.1007/s00795-020-00277-z)

Online Resource 2. Representative image of RCAS1, along with cell cycle markers for G1, S, and G2 phase

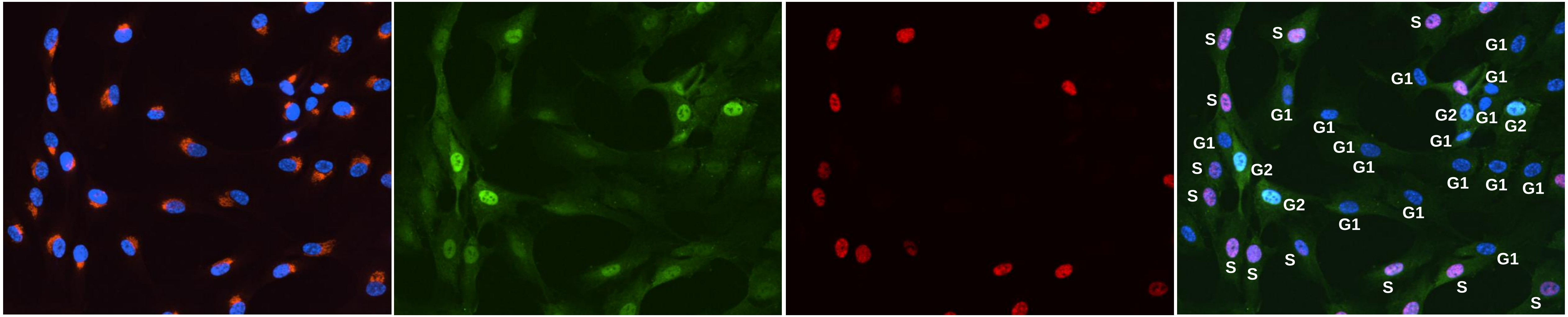

Supplement: Supplementary file 2 — Supplementary file2 (PDF 84 KB) [file 795_2020_277_MOESM2_ESM.pdf]
